# 2302: Downregulation of miR-1207-3p is correlated to upregulation of FNDC1, FN1, AR, and c-MYC in aggressive prostate cancer in men of African ancestry

**DOI:** 10.1017/cts.2017.37

**Published:** 2018-05-10

**Authors:** Dibash K. Das, Akintunde T. Orunmuyi, Gabriel Olabiyi Ogun, S. Adekola Adebayo, A. Ayo Salako, Adeodat Ilboudo, E. O. Olapade-Olaopa, Olorunseun O. Ogunwobi

**Affiliations:** Department of Biological Sciences, Hunter College, New York, NY, USA

## Abstract

OBJECTIVES/SPECIFIC AIMS: Prostate cancer is the second most common cancer in the world for men. For reasons still unclear, aggressive PCa disproportionately affects males of African ancestry (MoAA). Incidence and mortality rates are highest in MoAA as they have consistently shown a 2.3–3.0-fold higher risk of mortality compared with Caucasian men. This aggressiveness of PCa may be due to specific biological factors. Studies have established that microRNAs (miRNAs), essential in post-transcriptional gene regulation, are dysregulated in PCa. miR-1207-3p is encoded at the PVT1 gene locus, which is located downstream of MYC on the 8q24 PCa susceptibility locus. The chromosomal region 8q24 is associated with aggressive PCa. Yet miR-1207-3p in PCa in MoAA has never been investigated. Moreover, studies have shown that PVT1/MYC co-operation is a fundamental feature in all cancers with 8q24 amplification and 98% of the 8q24 amplicons contained concurrent amplification of the MYC and PVT1 loci. Moreover, MYC has been linked to PCa aggressiveness, and has been reported to be downstream of androgen receptor (AR) in some PCa. However, the mechanisms regulating c-MYC have never been studied in MoAA. We have recently demonstrated that miR-1207-3p has prognostic value in PCa, and directly binds to the 3′ UTR of Fibronectin type III domain containing 1 (FNDC1) to regulate a novel FNDC1/fibronectin (FN1)/AR pathway upregulated in metastatic PCa. However, the relevance of this novel and clinically significant miR-1207-3p molecular pathway in PCa in MoAA is unknown. Therefore, we hypothesized that miRNA 1207-3p, encoded at the 8q24 PCa susceptibility locus, is a PCa biomarker with clinical applications in MoAA. Our specific aim was to assess the clinical relevance of miR-1207-3p, FNDC1, FN1, AR, and MYC expression in aggressive PCa in a cohort of West African Black males. METHODS/STUDY POPULATION: Consequently, we aimed to determine the expression profile of miRNA-1207-3p, FNDC1, FN1, AR, and MYC in histologically confirmed normal prostate (n=24), benign prostate (n=44) and malignant prostate tissue (n=29) from prostatectomy or transrectal ultrasound-guided biopsies in patients recruited at the University College Hospital, Ibadan, Nigeria, a sub-Saharan Black African population. In total, 17 patients had tumor tissues with Gleason score ≥8. Tissues were collected in compliance with Institutional Ethics Board approved protocols. RNA extraction, cDNA synthesis, and quantitative-PCR were performed to analyze mRNA expression of miRNA-1207-3p, FNDC1, FN1, AR, and MYC. Statistical analysis were performed using SPSS software. All data were analyzed by the 1-way ANOVA test. Tukey post-hoc test was performed to determine the differences in mean expression between normal and tumor prostate tissues. *p*<0.05 were considered significant. RESULTS/ANTICIPATED RESULTS: We discovered that miR-1207-3p is significantly underexpressed in prostate tumor tissues [0.09±0.02, 95% CI (0.04, 0.136), *p*=0.000] in comparison with normal prostate tissue in MoAA [0.92±0.15, 95% CI (0.60, 1.244), *p*=0.000]. This is the first description of miR-1207-3p differential expression in human clinical PCa in MoAA. In contrast, FNDC1 was significantly overexpressed in prostate tumor tissues [21.93±8.21, 95% CI (4.97, 38.89), *p*=0.003] in comparison with normal prostate tissues in MoAA [1.57±0.45, 95% CI (0.625, 2.51), *p*=0.003]. The same positive correlation with advanced disease held true for FN1 [tumor: 13.66±3.53, 95% CI (6.38, 20.93), *p*=0.000; normal: 1.07±0.235, 95% CI (0.58, 1.56), *p*=0.000], AR [tumor: 20.49±6.74, 95% CI (6.50, 34.48), *p*=0.000; normal: 0.94±0.20, 95% CI (0.52, 1.36), *p*=0.000], and c-MYC [tumor: 33.93±8.43, 95% CI (16.53, 51.33), *p*=0.000; normal: 1.94±0.36, 95% CI (1.18, 2.70)]. The significantly increased mean expression for FNDC1, FN1, AR, and c-MYC in prostate tumor tissues in comparison with normal prostate tissues indicate that their overexpression is associated with increased risk of cancer progression in MoAA. DISCUSSION/SIGNIFICANCE OF IMPACT: These results show that the underexpression of miR-1207-3p and the overexpression of FNDC1, FN1, AR and MYC is associated with aggressive PCa in MoAA. miR-1207-3p, and it molecular target FNDC1, may be useful biomarkers for prognostication of PCa in MoAA.

